# Fruit and Vegetable Intake and Telomere Length in a Random Sample of 5448 U.S. Adults

**DOI:** 10.3390/nu13051415

**Published:** 2021-04-23

**Authors:** Larry A. Tucker

**Affiliations:** College of Life Sciences, Brigham Young University, Provo, UT 84602, USA; tucker@byu.edu; Tel.: +01-801-422-4927

**Keywords:** telomere, diet, carbohydrate, NHANES, antioxidant, inflammation, legume, potato

## Abstract

The relationship between fruit and vegetable intake and telomere length was examined using a cross-sectional design and an NHANES random sample of 5448 U.S. adults. Fruit and vegetable (F&V) consumption was assessed using a 24 h recall, and telomere length, an index of cellular aging, was measured using the quantitative polymerase chain reaction method. Telomere length was linearly related to F&V intake when combined (F = 22.7, *p* < 0.0001) and also when separated as fruit (F = 7.2, *p* < 0.0121) or vegetables (F = 15.4, *p* < 0.0005), after adjusting for covariates. Specifically, telomeres were 27.8 base pairs longer for each 100 g (3.5 ounces) of F&V consumed. Because each additional year of chronological age was associated with telomeres that were 14.9 base pairs shorter, when women and men were analyzed together, results indicated that a 100 g (3.5 oz) per day increment in F&V corresponded with 1.9 years less biological aging. When the 75th percentile of F&V intake was compared to the 25th, the difference was 4.4 years of cellular aging. When separated by sex, fruits and vegetables were both related to telomere length in women, but only vegetable intake was predictive of telomere length in men. In conclusion, evidence based on a random sample of U.S. adults indicates that the more the servings of F&V, the longer telomeres tend to be.

## 1. Introduction

There are many health benefits associated with vegetable and fruit intake. Unfortunately, approximately 85% of Americans do not consume sufficient amounts of vegetables and over 75% fall short regarding intake of fruits [1]. Consequently, the U.S. report, “Dietary Guidelines for Americans (2015–2020),” recommends that individuals increase their intake of vegetables from all vegetable subgroups. The guidelines also encourage Americans to shift toward consuming more fruits, mostly whole fruits, in nutrient-dense forms [1]. Similarly, the World Health Organization (WHO) recommends that adults consume more than 400 g (>14 oz) of fruits and vegetables per day to improve overall health and reduce the risk of disease [2]. 

Numerous investigations show that mortality decreases as consumption of vegetables and fruits increases. In a recent meta-analysis that included over 830,000 adults, Wang et al. combined 16 prospective cohort studies with follow-up periods ranging from approximately 5 years to 26 years [3]. Pooled results showed that all-cause mortality was 5–6% lower for each serving of fruits and vegetables, with a threshold of approximately five servings per day. Pooled findings for cardiovascular mortality were comparable [3]. 

Similarly, in a 2016 Australian cohort study of over 150,000 adults, Nguyen et al. showed that fruit and vegetable intake was associated significantly with reduced risk of all-cause mortality [4]. With an average follow-up period of 6.2 years, the investigation pointed out that the highest risk reduction was seen with seven servings of fruits and vegetables per day [4]. 

Vegetable and fruit intake could decrease mortality and reduce biologic aging by preserving telomeres. Telomeres are the DNA protein caps that provide stability and shield the ends of chromosomes [5]. When cells divide, a portion of the telomeric DNA does not replicate. Therefore, mitosis causes telomeres to gradually shorten. Because somatic cells experience a finite number of cell divisions, telomere length is highly related to chronological age [6]. It reflects a person’s telomere length when born and subsequent telomere breakdown. Hence, the shortening of telomeres is a mechanism and an index of cell aging [7,8].

Adults vary considerably within physiological, motor, cognitive, sensory, health, and other areas of function as chronological age increases. Vast differences exist within these domains even among adults of the same chronological age. Although chronological age is a good index of the health and function of adults, the length of telomeres can quantify biological aging beyond the reach of the calendar. 

Because telomeres shorten with the passage of time, are highly variable across individuals, are good predictors of a number of age-sensitive diseases and conditions, and are well-established within critical biological processes, telomere length is considered a meaningful measure of biological aging, as shown in a review by Mather et al. [9]. However, some studies indicate that telomere length is not a significant predictor of biological aging [10,11]. 

Evidence supporting the use of telomeres as an index of cell aging is plentiful. Cawthon et al. studied older adults for 15 years [12]. Results showed that individuals with shorter telomeres had 1.9-fold greater all-cause mortality compared to those with longer telomeres. Differences in mortality from heart disease was 3.2-fold greater for those with the shorter telomeres [12]. Similarly, in a prospective investigation by Bakaysa and associates [13], Swedish twins who had shorter telomeres compared to their co-twin had approximately 3-fold higher mortality over 7 years compared to the co-twin [13]. Finally, in a sample of almost 700 Italians, Ehrlenbach et al. found that shorter telomeres at baseline were predictive of greater all-cause mortality over 10 years [14]. Specifically, those who survived during the 10 years of follow-up had telomeres that were approximately 50% longer (median) than those who died. 

Scientists have investigated the relationship between telomere length and consumption of fruits and vegetables with varying results. In an investigation based on 455 normotensive men living in China, Lian et al. showed that higher vegetable intake was associated with longer telomeres [15]. Fruit intake was not related to telomere length, however. Marcon et al. uncovered similar findings [16]. Specifically, as vegetable intake increased, telomeres increased in length, but again, fruit consumption was not related to telomere length. However, Lee et al. studied 1958 middle-aged and older Korean adults and found the opposite [17]. These researchers showed that fruit intake was predictive of longer telomeres, but vegetable consumption was not. Conversely, Bethancourt et al. found that neither fruits nor vegetables were predictive of telomere length in 1459 young adult Filipino men [18]. These disparate findings do not appear to be a result of differences in statistical control of covariates. However, the mixed findings could be a result of differences in factors such as culture, lifestyle, and medical systems. 

The effect of fruits and vegetables on telomere length is not clear, especially in U.S. adults. To date, little research has focused on this research question using large samples of U.S. adults. Hence, the aim of this investigation was to evaluate the degree to which consumption of fruits and vegetables accounts for differences in telomere length in an NHANES sample of over 5000 men and women representing the adult population of the U.S. An ancillary aim was to evaluate the degree to which demographic and lifestyle variables influence the relationship between intake of vegetables and fruits and the length of telomeres. Lastly, vegetable intake was defined using multiple definitions. Specifically, vegetable consumption was studied with and without legumes included, and with and without the inclusion of potatoes. 

## 2. Materials and Methods

The present investigation was conducted employing a cross-sectional design and data collected as part of the ongoing National Health and Nutrition Examination Survey (NHANES) in the United States. Overseen by the U.S. Centers for Disease Control and Prevention, NHANES has been conducted for many decades to provide ongoing estimates of the health and nutrition status of non-institutionalized individuals living in the United States. A multifaceted sampling design is employed by NHANES to enable the findings to be generalized throughout the United States [19]. Specifically, NHANES uses a four-stage sampling design with random selection employed at each stage. First counties are selected, followed by city blocks, then households are chosen, and lastly individuals are selected for participation [19].

The length of leukocyte telomeres was measured by NHANES during a four-year period in the United States only, 1999–2002. During this four-year period, multiple subcategories were oversampled to afford more exact estimates, including low-income individuals, Mexican Americans, Non-Hispanic Black Americans, individuals ages 60 or older, and teenagers 12–19 years old [19].

For the present study, adults were asked to give a blood sample for DNA analysis. A total of 76% consented and gave a useable sample. To maximize confidentiality, NHANES assigned all adults 85 years old and older the age of 85. Hence, participants that were 85 years or older were excluded from the sample. Subjects with missing data or extreme results (±3 standard deviations from the mean) were also excluded from the sample [20,21,22,23]. A total of 5448 adults, 2935 women and 2647 men, were included in the analyses. Written informed consent was acquired from each participant. The Ethics Review Board (ERB) approved the data collection protocol. The ethical approval code for NHANES data collection from 1999–2002 was #98-12 [24]. 

### 2.1. Measures

More than a dozen variables were measured in the present study: fruit intake, vegetable consumption (including and excluding potatoes, and including and excluding legumes and pulses), estimated energy intake, leukocyte telomere length, age, sex, race, body mass index (BMI), smoking, physical activity, and alcohol use. 

#### 2.1.1. Fruits and Vegetables

Dietary assessments were performed in private settings within an NHANES Mobile Examination Center (MEC). A computer-assisted procedure, managed by a qualified NHANES interviewer, was employed to gather the dietary data [25]. A standardized interview protocol was followed for each participant [26]. Food probes were part of the interview format. All the interviewers spoke at least two languages and each had a degree in nutrition or home economics. Each dietary assessment focused on foods and beverages consumed during the previous 24 h (midnight to midnight). Numerous investigations have employed the 24 h recall system managed by NHANES to collect data on dietary intake in adults [27,28,29]. 

Concurrent validity of the NHANES 24 h diet assessment has been established through numerous studies. For example, results derived using the NHANES 24 h recall are predictive of non-alcoholic fatty liver disease [30], total mortality [31], cardiovascular mortality [32], cancer mortality [33], and coronary heart disease [34], to name a few.

Consumption of fruits and vegetables served as the exposure variables of the present study. Fruit intake was measured in grams. Fruit drinks and juices were not counted as part of the fruit intake total. Vegetable consumption, cooked or raw, was also measured in grams, and included starchy vegetables. However, in separate analyses, potatoes and potato soups were excluded from the vegetable group, as recommended by the World Health Organization [2] and a number of independent investigations [35,36,37]. Similarly, consumption of legumes and pulses were analyzed as part of the vegetable group and also separately. According to the United Kingdom Eatwell Plate program, pulses are not a standard vegetable [38]. The Eatwell Plate program states, “…while pulses contain fibre, they don’t give the same mixture of vitamins, minerals and other nutrients as fruit and vegetables” [38].

Intake of fruits and vegetables was reported separately and combined. Additionally, because intake of specific foods tends to increase as energy intake increases, two methods were employed to quantify fruit and vegetable consumption. First, intake was expressed simply as grams consumed per day, not accounting for differences in energy intake. Second, intake of fruits and vegetables was standardized based on energy intake. In short, fruit and vegetable intakes were expressed as grams consumed per 1000 kcal.

#### 2.1.2. Energy Intake

Underreporting energy or kilocalorie (kcal) intake is a common problem when food consumption is self-reported [39]. Hence, the Mifflin resting metabolic rate formula (RMR) [40], combined with a measure of physical activity level (PAL) [41], were used to estimate energy expenditure and energy intake [42]. PAL was assessed using four NHANES questions, each describing a higher level of physical activity [41]. Each of the four levels of physical activity was assigned a PAL value: 1.45, 1.55, 1.65, and 1.75, respectively. Multiplied together, resting metabolic rate and physical activity level were used to approximate total energy consumption, as reported in other research [43].

#### 2.1.3. Telomere Length

The outcome variable of the present study was leukocyte telomere length, an indicator of the biological age of humans. Leukocyte telomeres are the most commonly used telomeres in research. From time to time, other tissues are used, such as colon, skin, nerves, muscle, and mucosa, but these tissues are much less common for the study of telomeres. According to Demanelis et al., variations in the lengths of telomeres based on tissue types, and the extent of the associations between tissue type telomere lengths can be ascribed to differences in both internal (e.g., cell division rate and history, telomere maintenance) and external (e.g., response to environmental exposures) factors across tissues [44]. Telomeres derived from blood (leukocytes) are used most often because they are the best all-around substitute for telomeres in other tissues [44]. 

The length of telomeres was evaluated with care to minimize measurement error, as described by NHANES and others [45,46,47]. The interassay coefficient of variation was 6.5% [47]. The relative telomere to single copy gene (T/S) ratios were transformed to base pairs using the formula: 3274 + 2413 × (T/S) [47]. 

#### 2.1.4. Weight and Height

A digital scale was employed to assess body weight. During the measurement, the subject wore only underclothes, a disposable paper gown, and foam slippers [48]. A mounted stadiometer with an adjustable headboard was employed to assess height [48]. Weight and height were utilized to figure body mass index (BMI), defined below. 

#### 2.1.5. Sociodemographic Covariates

There were three sociodemographic covariates included in the present study: age, sex, and race. Chronological age ranged from 20 to 84 years. To maximize confidentiality, all participants older than 85 years were recorded as 85 by NHANES, so individuals older than 84 were not included in this study. NHANES defined sex using two categories, female or male. NHANES defined race and ethnicity using five categories: Non-Hispanic White, Non-Hispanic Black, Mexican American, Other Race or Multi-Racial (Other), and Other Hispanic. 

#### 2.1.6. Lifestyle Covariates

Four lifestyle factors were used as covariates and were controlled statistically in some models: BMI, alcohol use, total physical activity, and smoking. BMI was employed to index body weight independent of height. BMI was determined employing the formula: weight in kilograms divided by height in meters squared, kg/m^2^ [49]. Categories based on standard cut-off points were used: underweight (<18.5), normal weight (≥18.5 and <25.0), overweight (≥25.0 and <30.0), obese (≥30.0), or missing. 

Alcohol consumption was indexed using three categories—abstainers, moderate drinkers, and heavy drinkers—as described by NHANES [50]. The NHANES question (ALQ130) was used to assess the number of alcoholic drinks consumed by participants. 

To assess participation in physical activity (PA), MET (metabolic equivalent) minutes per week during the past 30 days were calculated. Adults indicated their involvement in 62 separate PAs, if any, and if the activity was moderate or vigorous in intensity, frequency of participation over the past 30 days, and the amount of time spent in the PA. Less than 10 min of PA was counted as 0. A MET score was estimated for each PA reported and total MET-min per week were estimated by NHANES for each adult by employing the physical activity compendium [51]. 

Smoking was indexed using pack-years, which was calculated as the number of cigarettes smoked per day multiplied by the years smoked, divided by 20 [52]. 

### 2.2. Statistical Analysis

NHANES participants are unique because they are selected randomly from the U.S. adult population. Each participant is assigned an individual sample weight, allowing that person to represent other individuals with similar characteristics. Statistical outcomes were founded on the sophisticated sampling design of NHANES, which included strata, clusters, and sample weights. Hence, statistical results reported in this paper represent the civilian, non-institutionalized, adult population of the United States.

Although the sample size of this investigation was large (*n* = 5448), degrees of freedom (df) were small in comparison. Because a multi-stage sampling design was employed by NHANES, degrees of freedom for each analysis were computed as the number of clusters (*n* = 57) minus the number of strata (*n* = 28), or 29 df [53]. 

SAS SurveyMeans was used to compute weighted means and SurveyFreq was used to estimate weighted frequencies, each generalizable to the U.S. population. Fruit and vegetable intakes were each indexed using two variables, intake in grams, and grams consumed per 1000 kcal. The extent of the linear relationships between fruit, vegetable, potato, and legume intakes and telomere length were calculated using regression analysis and the SAS SurveyReg procedure [53]. Regression estimates were adjusted based on sampling weights. The SAS SurveyReg procedure and partial correlation were utilized to determine the degree that associations between fruit and vegetable consumption and telomere length were influenced by the covariates [53]. Regression coefficients were used to calculate the number of telomere base pairs associated with each year of age, and to determine the number of base pairs associated with each serving of F&V. Statistical significance was based on *p* < 0.05. The statistical analyses were conducted employing SAS Version 9.4 (SAS Institute, Inc., Cary, NC, USA).

## 3. Results

There were 5448 adults in the sample. Mean age (±SE) of the sample was 46.5 (±0.4) years. Mean telomere length was 5824 (±39) base pairs, average fruit intake was 78.9 (±2.8) grams per day, and mean vegetable consumption (without potatoes or legumes) was 93.8 (±3.2) grams per day. Potato intake averaged 50.6 (±1.4) grams per day, and consumption of legumes and pulses averaged 24.2 (±1.7) grams per day. Mean (±SE) estimated energy intake was 2410 (±12) kilocalories per day. Table 1 shows the mean (±SE) and percentile values of the continuous variables of the investigation when all the participants were included.

In the present study, sex, race, BMI, and alcohol use were treated as categorical variables. Of the 5448 participants, 52.9% (±0.7) were female, 70.2% (±1.9) were Non-Hispanic White, 12.1% (±1.4%) were Non-Hispanic Black, 7.6% (±0.9) were Mexican American, 3.6% (±0.6%) placed themselves within the category labeled Other Race or Multi-Racial, and 6.5% (±1.6%) identified themselves as Other Hispanic. For the BMI categories, 31.7% (±1.1) were obese, 34.0% (±1.1) were overweight, 31.5% (±0.8) fit within the normal category, 2.0% (±0.3) were considered underweight, and 0.8% (±0.2) had missing data. For the alcohol use variable, 35.5% (±2.7) indicated that they were alcohol abstainers, 32.2% (±1.7%) were labeled moderate drinkers, and 32.3% (±1.4) were found to be heavy drinkers. 

### 3.1. Age and Telomere Length 

Age and telomere length were inversely related (F = 421.0, R^2^ = 0.156, r = −0.39, *p* < 0.0001). For each year of age, telomeres were 14.9 base pairs shorter, determined using the regression coefficient. Beyond the linear term, age^2^ was unrelated to telomere length (F = 0.1, *p* = 0.8234). Adjusting statistically for age^2^ separately or combined with age had no effect on the relationship between fruit and vegetable intake and telomere length.

### 3.2. Intake of Fruits and Vegetables Combined and Telomeres

As displayed in Table 2, with data from women and men combined, telomere length was linearly related to fruit and vegetable (F&V) intake (F = 22.7, *p* < 0.0001). After adjusting for differences in age, sex, and race, telomeres were 27.8 base pairs longer for each 100 g (3.5 ounces) of F&V consumed. After controlling for the sociodemographic covariates (age, sex, and race) and the lifestyle covariates (BMI, physical activity, alcohol use, and smoking pack-years), telomeres were 24.7 base pairs longer for each 100 g (3.5 ounces) of F&V consumed (F = 20.3, *p* < 0.0001). 

When F&V intake was expressed as grams consumed per 1000 kcal, the relationship was stronger than when calculated simply as grams eaten (F = 25.9, *p* < 0.0001). Specifically, after adjusting for differences in the sociodemographic covariates, for each F&V consumption increment of 100 g (3.5 ounces) per 1000 kcal, telomeres were 66.9 base pairs longer. With all the covariates controlled, telomeres were 57.6 base pairs longer, on average (F = 23.3, *p* < 0.0001). 

### 3.3. Fruit Intake and Telomeres

With data from women and men combined, fruit intake was related significantly and positively to leukocyte telomere length. With age, sex, and race controlled statistically, for each 100 g (3.5 ounces) of fruit consumed, telomeres were 26.9 base pairs longer, on average (F = 7.2, *p* = 0.0121). After adjusting for differences in the sociodemographic covariates (age, sex, race) and the lifestyle covariates (physical activity, smoking, BMI, and alcohol use), the association between fruit intake and telomere length remained significant and positive. Specifically, for each 100 g (3.5 ounce) higher intake of fruits, telomeres were 23.1 base pairs longer (F = 6.0, *p* = 0.0206), on average. 

Instead of expressing fruit intake as total grams consumed, reporting grams consumed per 1000 kcal generally strengthened the relationship between fruit intake and telomere length. After adjusting for the three sociodemographic covariates, for each 100 g (3.5 ounces) consumed per 1000 kcal, telomeres were 74.9 base pairs longer, on average (F = 10.8, *p* = 0.0027). After controlling for the sociodemographic and lifestyle covariates together, the association remained strong and significant (F = 8.7, *p* = 0.0063). Specifically, for each 100 g (3.5 ounces) of fruit eaten per 1000 kcal, telomeres were 63.1 base pairs longer, on average (Table 2). 

### 3.4. Vegetable Intake and Telomeres

Vegetable intake, especially with potatoes excluded, was a significant predictor of telomere length, with data from women and men analyzed together. Telomeres were 32.5 base pairs longer for each 100 g (3.5 ounces) of vegetables consumed, after controlling for age, sex, and race (F = 15.4, *p* = 0.0005). Further, they were 28.9 base pairs longer after adjusting for all the covariates simultaneously (F = 13.2, *p* = 0.0011). With age, sex, and race controlled, telomeres were 71.4 base pairs longer for each 100 g (3.5 ounces) per 1000 kcal consumed (F = 25.9, *p* < 0.0001). Similarly, with the lifestyle covariates controlled, along with the sociodemographic covariates, telomeres were 59.1 base pairs longer for each 100 g (3.5 ounces) per 1000 kcal consumed (F = 21.3, *p* < 0.0001). 

### 3.5. Fruit and Vegetable Intake

Fruit intake and vegetable consumption were positively related to each other. Specifically, with no covariates in the model, fruit intake accounted for 2.9% of the variance in vegetable intake (*p* < 0.0001) and vice versa. After adjusting for differences in age, sex, and race, the fruit and vegetable relationship demonstrated 2.5% overlapping variance (*p* < 0.0001). Both fruit intake (F = 5.0, *p* = 0.0341) and vegetable consumption (F = 12.1, *p* = 0.0016) were predictive of telomere length when placed into the same competitive regression model.

### 3.6. Intake of Legumes and Potatoes and Telomeres

As shown in Table 2, none of the relationships between legume and pulse consumption and telomere length were significant. Additionally, all the relationships between potato intake and telomere length were inverse and none were significant. However, when vegetable intake included potato consumption, the relationship with telomere intake remained positive and significant, although weaker than when vegetable intake excluded potato consumption. Similarly, when vegetable consumption included legume intake, the relationship was weakened, but it remained statistically significant.

### 3.7. Fruits, Vegetables, and Telomeres in U.S. Women Only

Table 3 shows the relationships between fruit and vegetable consumption, with and without potato and legume intake, and telomere length in U.S. women. With fruit and vegetable intakes combined, women with the highest intakes had the longest telomeres. Fruit intake was predictive of telomere length with age and race controlled (model 1) and also after adjusting for the other covariates, model 2. With vegetable intake expressed as grams per 1000 kcal, intake was positively associated with telomere length, in both models 1 and 2. However, when expressed as grams of vegetables consumed (not per 1000 kcal), intake was related to telomeres in model 1 but not in model 2. In U.S. women, vegetable intake, when combined with potato consumption, was positively associated with telomere length. Potato and legume intakes, when considered separately, were not predictive of telomere length in U.S. women, as shown in Table 3. 

### 3.8. Fruits, Vegetables, and Telomeres in U.S. Men Only

Table 4 displays the associations between fruit and vegetable intake, without and with legume intake and potato consumption, and telomere length in U.S. men only. In all models, fruit and vegetable consumption, when combined, was associated with telomere length. The significant relationships persisted whether or not the sociodemographic or the sociodemographic and lifestyle covariates were controlled. Similarly, vegetable consumption was significantly and positively related to telomere length in models 1 and 2, as displayed in Table 4. When potatoes were combined with vegetables, the relationship was significant in model 1 but not in model 2. Likewise, when potatoes and legumes were combined with vegetable consumption, intake levels were not associated with telomere length. Additionally, unlike U.S. women, fruit intake was not predictive of telomere length within any of the models in U.S. men, except when combined with vegetable consumption (F&V). Lastly, potato intake and legume consumption, when considered separately, were not related to telomere length in any of the models in U.S. men.

## 4. Discussion

The principal goal of this investigation was to ascertain the association between fruit and vegetable (F&V) intake and leukocyte telomere length in a randomly selected sample of 5448 women and men who represented the non-institutionalized, civilian U.S. adult population. An ancillary aim was to evaluate the associations between potato consumption, and legume intake, and telomere length. Another objective was to assess the extent to which a number of sociodemographic and lifestyle covariates affected the relationships between fruit and vegetable intake and telomere length. 

There were six key findings in this investigation. First, using the data for women and men combined, the relationship between F&V intake and telomere length was significant, positive, and meaningful, whether fruits and vegetables were evaluated together or separately. Second, consumption of potatoes, when considered separate from other vegetables, was not related to telomere length. Third, legume and pulse intake, when analyzed separate from other vegetables, was not related to the length of telomeres. Fourth, statistical control of the sociodemographic and lifestyle covariates had little effect on the relationships. Fifth, when the sample was delimited to U.S. women only, fruit intake and vegetable consumption were each related to biological aging. Sixth, when focusing on U.S. men only, vegetable intake was positively associated with telomere length, but fruit consumption was not. 

In general, the more F&V consumed, the longer the telomere length tended to be. Intake of fruits and vegetables was directly related to telomere length. The associations were linear. After adjusting for differences in all the covariates (age, sex, race, smoking, physical activity, alcohol use, and BMI), regression analysis showed that each 100 g (3.5 ounce) increment of F&V consumption (combined) was predictive of telomeres that were 24.7 base pairs longer, on average (Table 2). Given each year of chronological age was associated with leukocyte telomeres that were 14.9 base pairs shorter, on average, each increment of 100 g (3.5 ounces) was associated with 1.7 fewer years of biological aging (24.7 ÷ 14.9 = 1.7 years).

Focusing on F&V intake combined, the U.S. 75th percentile (264 g) and the 25th percentile (0 g) differed by 264 g. Given each 100 g (3.5 ounce) increment in consumption was linked to telomeres there were 24.7 base pairs longer (Table 2), and given each 14.9 base pair differential was associated with 1.0 year of cellular aging, the difference between the 75th and 25th percentiles represented a difference of approximately 4.4 years of biological aging (100 g F&V = 24.7 base pairs; difference between the 75th and 25th percentiles = 264 g of F&V; 264 g ÷ 100 g = 2.64; 24.7 base pairs × 2.64 = 65.2 base pairs; 65.2 ÷ 14.9 = 4.4 years). Most would consider a biological aging difference of 4.4 years meaningful. 

Level of consumption of cooked and raw vegetables (excluding potatoes and legumes) was a good predictor of biological aging. After controlling for all the covariates, for each 100 g of vegetables consumed, telomere base pairs were 28.9 base pairs longer, on average. Interpretation of these findings by comparing the 75th (146 g) and 25th (0 g) percentiles of intake indicated that adults at the 75th percentile had approximately 2.8 fewer years of biological aging, on average, than those at the 25th percentile of vegetable intake (146 ÷ 100 = 1.46; 1.46 × 28.9 = 42.2; 42.2 ÷ 14.9 = 2.8 years).

Intake of fruits was also a predictor of telomere length in the combined sample. The more fruits that were consumed, the longer the telomeres tended to be. After adjusting for all the covariates, for each 100 g (3.5 ounces) of fruits eaten, telomere base pairs were 23.1 base pairs longer, on average. Interpretation of these findings by comparing the 75th (130.9 g) and 25th (0 g) percentiles of intake showed that adults at the 75th percentile had approximately 2.0 fewer years of cellular aging, on average, than those at the 25th percentile of fruit consumption (130.9 ÷ 100 = 1.31; 1.31 × 23.1 = 30.3; 30.3 ÷ 14.9 = 2.0 years).

Each F&V consumption variable was expressed using two forms: (1) grams, (2) grams per 1000 kcal. Intake based on grams was straightforward. However, consumption was also standardized based on energy intake (g per 1000 kcal) to help offset the fact that adults who consume more total kilocalories tend to eat more fruits and vegetables. Indexing intake based on energy consumption allowed intake to be viewed as a proportion of total consumption (relative intake), rather than absolute intake (grams). In the present study, the relationships between F&V intake and telomere length were generally stronger when intake was standardized based on energy intake (g per 1000 kcal) compared to when the energy intake of the participant was ignored (grams).

A number of investigations have studied the link between intake of various foods and their relationships with telomere length. For example, consumption of sugar-sweetened soda was predictive of shorter telomeres. Specifically, for each 8 ounce serving per day, adults had 1.8 years of increased cellular aging, on average [54]. Similarly, for each 200 kcal of nuts and seeds consumed per day, adults had 1.7 years of decreased biological aging [55]. Furthermore, when adults with the highest quartile of fiber intake were compared to those in the lowest quartile, the cellular aging difference was almost 5 years [6]. Given the results of the present study, consumption of fruits and vegetables seems to compare inversely with sugar-sweetened soda, and positively with nuts and seeds, and dietary fiber, for potential protection against biological aging.

F&V consumption, treated separately or combined, was linearly related to telomere length in the present study. However, intake of particular foods does not happen in isolation. For example, in this investigation, adults who ate significant amounts of fruits and vegetables probably also ate higher levels of fiber and whole grains [1,56]. Fiber and whole grain intakes are connected to reduced risk of mortality and disease [57,58]. Consumption of fruits and vegetables is also associated with decreased disease and mortality [1,59]. Diets high in fiber and whole grains, and diets with large amounts of fruits and vegetables, tend to go hand-in-hand [56]. In short, some of the aging benefits associated with eating large amounts of fruits and vegetables, as seen in this study, might be partly a result of consuming higher levels of fiber and whole grains and perhaps other healthy foods.

When separated by sex, the relationships between both fruit and vegetable intakes and biological aging remained significant for women. However, for men, vegetable consumption was related to telomere length, but fruit intake was not. Why there was a difference between men and women is not clear. In both groups, differences in vegetable intake accounted for more variance in telomere length than fruit consumption. 

Adjusting statistically for differences in age, sex, and race, reduced the likelihood that these sociodemographic factors influenced the results. Additionally, controlling for differences in physical activity, smoking, alcohol use, and BMI, minimized the influence of these lifestyle covariates. In general, controlling statistically for the lifestyle factors, in addition to the sociodemographic variables, weakened the relationship between fruits and vegetables and telomere length by an additional 15%. With all the covariates controlled, the findings showed the association between F&V intake and biological aging, as if all participants had the same age, sex, race, physical activity level, smoking habit, alcohol drinking behavior, and body mass index. Hence, it appears that only a small portion of the association between F&V and biological aging can be attributed to differences in these lifestyle factors. 

In 2017, Rafie et al. reviewed several dietary studies that focused on the association between various foods, groups of foods, and patterns of eating, and telomere length [60]. Results were not consistent. A total of 13 investigations in the review targeted the relationship between fruit and/or vegetable intake and telomere length [60]. Five of the studies reported significant, positive associations. However, the other eight investigations failed to find significant relationships between fruits and/or vegetables and biological aging. The mixed results could be partly due to the varying methods used to classify fruits and vegetables, especially the latter. The current study is a good example. When potato intake was included as part of the vegetable group, the relationship was weakened significantly. In fact, when isolated, the potato food group was inversely associated with telomere length and the relationship was not significant. Similarly, the legume food group, which is sometimes considered part of the vegetable group, was not significantly related to biological aging when isolated from the vegetable group. In the present investigation, when the vegetable group was analyzed with both potatoes and legumes included, the relationship with telomere length was attenuated substantially, but it remained significant. Given the results of the present study, when developing a dietary pattern most predictive of reduced biologic aging, potatoes and legumes should probably not be included with the vegetable group. 

Why were higher consumption levels of fruits and vegetables predictive of less biological aging (i.e., longer telomeres)? Although the exact mechanism is not known, it is likely that reduced inflammation and oxidative stress account for many of the differences [61,62,63,64]. Research indicates that telomere length serves as an index of the cumulative oxidative stress and inflammation of progenitor cells [5,65]. In other words, oxidative stress shortens telomeres [66]. On the other hand, F&V intake seems to preserve telomeres and reduce cell aging by reducing inflammation and oxidative stress. The benefits are not limited to adults. Research by Garcia-Calzon et al. shows that total dietary antioxidant capacity is related to leukocyte telomere length in children and adolescents [67]. 

Several systematic reviews indicate that fruit and vegetable intake reduces established markers of inflammation and oxidative stress [68,69,70,71]. Zhu cites numerous studies showing that phenolics in fruits and vegetables are the principal bioactive agents known to benefit health [70]. Moreover, in a meta-analysis by Hosseini et al., fruit and vegetable intake significantly reduced several inflammatory biomarkers, particularly tumor necrosis factor-alpha and C-reactive protein (CRP) [68]. Fruit and vegetable consumption was also associated with higher levels of gamma delta T cells (γ δ-T cells). Additionally, numerous investigations discussed by Hosseini et al. indicate that F&V intake positively affects immune cell function [68]. Independent of vegetable consumption, fruit intake also seems to have valuable effects on markers of systemic inflammation, although some studies have reported no effects [68,71].

In summary, fruits and vegetables include high levels of phytochemicals, which have significant antioxidant and anti-inflammatory properties [68,69,70,71]. By increasing antioxidants and anti-inflammatory levels in the body, F&V likely slow the biological aging process, which is manifested by longer leukocyte telomeres. Potato and legume intakes may be less related to telomere length compared to other vegetables because they seem to contain fewer phytochemicals and antioxidant properties [2,38]. Additionally, although speculative, potatoes may be unrelated to telomere length because they are often prepared with or garnished with a significant amount of fat, particularly saturated fat, in the United States. In short, it may not be the potatoes but the condiments they are consumed with that result in no relationship with telomere length. 

There were multiple limitations associated with the current study. Perhaps of most importance, because NHANES used a cross-sectional design, cause-and-effect inferences cannot be made. Second, adults who indicated that they consumed lots of F&V could represent adults who have behaviors that are healthier than others. As a result of this potential problem, adjustments were made statistically to control for potential mediating factors, including sociodemographic and lifestyle variables. These covariates had little impact on the key associations. However, other variables, not assessed in this investigation, could account for some of the significant relationships unveiled in this investigation. Additionally, energy intake was estimated. Use of doubly labeled water would have been a better method to estimate energy intake, but it was not available. Additionally, the present investigation used telomere length as a single measure of biological aging. Other indices of aging were not evaluated. Lastly, telomerase activity was not assessed. 

This study also possessed multiple strengths. For example, the sample was large, including almost 5500 adults of all races. Additionally, the age range was broad, including adults 20 to 84 years old. Second, because subjects were randomly selected, results are representative of the non-institutionalized, adult population of the U.S. Third, many sociodemographic and lifestyle measures were statistically adjusted for, reducing their influence on the associations of interest. Fourth, a high-quality laboratory was used to assess the length of leukocyte telomeres. In short, valid and reliable procedures were employed to generate the telomere information. Consequently, chronological age was a meaningful predictor of telomere length, as one would expect. 

Although the present investigation noted a significant relationship between fruit and vegetable consumption and longer telomeres in U.S. women, and vegetable intake and less biological aging in U.S. men, there remains much to learn about the relationships. To date, most research focusing on diet and telomere length has been cross sectional. Although long-term randomized controlled trials studying changes in telomeres are not feasible, prospective cohort studies would be valuable. Prospective telomere investigations lasting decades would help to determine whether abundant fruit and vegetable consumption reduces risk of developing short telomeres. 

## 5. Conclusions

Consumption of fruits and vegetables, considered separately and combined, was linearly related to telomere length in a large, random sample of women and men, when considered together. After adjusting for a number of sociodemographic and lifestyle covariates, for each 100 g (3.5 ounces) of fruit eaten per day, telomeres reflected 1.6 fewer years of biological aging. Similarly, for each 100 g (3.5 ounces) of vegetables consumed per day (excluding potatoes and legumes), the length of telomeres signified 1.9 fewer years of cellular aging. Comparing the U.S. 75th percentile to the 25th percentile of fruit and vegetable intake was predictive of 4.4 years of reduced cellular aging. On the other hand, potato intake and consumption of legumes and pulses were not related to telomere length. When the sample was delimited to U.S. women, both fruits and vegetables were inversely associated with biological aging. However, in U.S. men, vegetable intake was related to telomere length, but fruit consumption was not. Overall, this investigation highlights the higher levels of biological aging associated with adults who do not eat significant amounts of fruits and vegetables, especially the latter. Results of this study support the Dietary Guidelines for Americans (2015–2020), which encourage adults to eat large amounts of fruits and vegetables each day as part of a high-quality diet. 

## Figures and Tables

**Table 1:** Descriptive characteristics of the key variables in U.S. women and men (*n* = 5448).

		Percentile			
Variable	Mean	SE	25th	50th	75th
Age (years)	46.5	0.4	33.0	44.4	58.0
Telomere length (base pairs)	5824	38.9	5380	5743	6185
Body mass index (BMI)	28.3	0.2	23.7	27.1	31.6
Fruit intake (g)	78.9	2.8	0	0	130.9
Fruit intake (g per 1000 kcal)	35.0	1.4	0	0	55.3
Vegetable intake (g) ^†^	93.7	3.2	0	43.1	146.0
Vegetable intake (g per 1000 kcal) ^†^	40.9	1.4	0	18.2	61.7
Fruit and vegetable intake (g)	169.3	5.1	0	117.8	264.0
F&V intake (g per 1000 kcal)	74.9	2.2	0	47.3	113.6
Potato intake (g)	50.6	1.4	0	0	74.9
Potato intake (g per 1000 kcal)	20.7	0.6	0	0	30.1
Legume intake (g) ^‡^	24.2	1.7	0	0	0
Legume intake (g per 1000 kcal) ^‡^	10.3	0.7	0	0	0
Veg, potato, and legume intake (g)	169.4	3.7	22.3	120.5	252.9
Veg, potato, and legume intake (g per 1000 kcal)	72.2	1.7	9.2	49.9	109.8
Body weight (kg)	80.6	0.5	65.8	78.1	91.9
Physical activity (MET-min)	132.9	11.7	0	0	135.8
Energy intake (kilocalories)	2410	11.9	2035	2383	2742
Smoking (pack-years)	2.9	0.2	0	0	0

^†^ The vegetable food group did not include potatoes or legumes or pulses unless otherwise stated. ^‡^ Legume and pulse intake not combined with any other vegetable. F&V = fruit and vegetable intake combined. Veg = vegetables. MET-min was MET-minutes of PA per week. The dietary intake results reflect consumption per 24 h.

**Table 2:** Relationship between fruit and vegetable intake and telomere length in U.S. women and men combined.

Exposure Variable	Model	Regression Coefficient	SE	R^2^ (%)	F	*p*
Fruit and vegetable intake (100 g) ^†^	1	27.8	5.8	16.8	22.7	<0.0001
	2	24.7	5.5	17.5	20.3	<0.0001
Fruit a vegetable intake (100 g per 1000 kcal) ^†^	1	66.9	13.1	16.8	25.9	<0.0001
	2	57.6	11.9	17.4	23.3	<0.0001
Fruit intake (100 g)	1	26.9	10.1	16.4	7.2	0.0121
	2	23.1	9.5	17.2	6.0	0.0206
Fruit intake (100 g per 1000 kcal)	1	74.9	22.8	16.6	10.8	0.0027
	2	63.1	21.5	17.3	8.7	0.0063
Vegetable intake (100 g) ^†^	1	32.5	8.3	16.6	15.4	0.0005
	2	28.9	8.0	17.4	13.2	0.0011
Vegetable intake (100 g per 1000 kcal) ^†^	1	71.4	14.0	16.6	25.9	<0.0001
	2	59.1	12.8	17.3	21.3	<0.0001
Potato intake (100 g)	1	−5.8	8.6	16.2	0.5	0.5038
	2	−5.1	9.0	17.2	0.3	0.5732
Potato intake (100 g per 1000 kcal)	1	−6.8	22.9	16.2	0.1	0.7688
	2	−9.8	24.0	17.1	0.2	0.6861
Legume and pulse intake (100 g)	1	2..4	12.8	16.2	0.0	0.8533
	2	−0.6	13.1	17.0	0.0	0.9629
Legume and pulse intake (100 g per 1000 kcal)	1	5.0	28.5	16.1	0.0	0.8627
	2	−5.6	29.2	17.0	0.0	0.8498
Vegetable and potato intake (100 g)	1	16.9	4.9	16.4	12.1	0.0016
	2	14.9	4.6	17.2	10.6	0.0029
Vegetable and potato intake (100 g per 1000 kcal)	1	50.2	11.5	16.4	19.2	0.0001
	2	41.1	10.5	17.2	15.3	0.0005
Veg., potato, and legume intake (100 g)	1	15.1	4.7	16.4	10.2	0.0033
	2	12.4	4.5	17.2	7.5	0.0107
Veg., potato, and legume intake (100 g per 1000 kcal)	1	34.7	10.7	16.2	10.5	0.0030
	2	25.5	10.7	17.1	5.7	0.0235

^†^ Vegetable intake did not include potato or legume consumption unless otherwise noted. Model 1 included statistical adjustment for differences in age, sex, and race. Model 2 included adjustment for differences in age, sex, race, BMI, physical activity, smoking, and alcohol use. R^2^ represents the variance accounted for by the full model. Interpretation is as follows: Regarding fruit consumption, for each 100 g (3.5 ounce) higher intake of fruit, after adjusting for differences in age, sex, and race, telomeres were 26.9 base pairs longer, on average (F = 7.2, *p* = 0.0121).

**Table 3:** Relationship between fruit and vegetable intake and telomere length in U.S. women only.

Exposure Variable	Model	Regression Coefficient	SE	R^2^ (%)	F	*p*
Fruit and vegetable intake (100 g) ^†^	1	33.6	7.8	15.3	18.7	0.0002
	2	28.8	7.4	16.3	15.1	0.0005
Fruit and vegetable intake (100 g per 1000 kcal) ^†^	1	75.5	16.7	15.5	20.5	<0.0001
	2	63.5	16.3	16.3	15.2	0.0005
Fruit intake (100 g)	1	45.3	13.9	15.2	10.6	0.0029
	2	39.9	12.3	16.2	10.5	0.0030
Fruit intake (100 g per 1000 kcal)	1	100.8	28.3	15.4	12.7	0.0013
	2	85.8	25.1	16.3	11.6	0.0019
Vegetable intake (100 g) ^†^	1	27.7	13.2	14.7	4.4	0.0449
	2	21.5	13.7	15.8	2.5	0.1274
Vegetable intake (100 g per 1000 kcal) ^†^	1	50.6	17.6	14.8	8.3	0.0074
	2	33.5	17.0	15.8	3.9	0.0583
Potato intake (100 g)	1	1.1	19.2	14.4	0.0	0.9525
	2	4.2	21.0	15.6	0.0	0.8436
Potato intake (100 g per 1000 kcal)	1	22.0	42.7	14.4	0.3	0.6094
	2	20.8	46.6	15.6	0.2	0.6588
Legume and pulse intake (100 g)	1	16.3	16.9	14.4	0.9	0.3424
	2	13.7	18.2	15.6	0.6	0.4578
Legume and pulse intake (100 g per 1000 kcal)	1	28.2	33.7	14.4	0.7	0.4087
	2	18.3	35.2	15.6	0.3	0.6065
Vegetable and potato intake (100 g)	1	21.2	7.9	14.7	7.1	0.0123
	2	17.9	7.9	15.8	5.1	0.0315
Vegetable and potato intake (100 g per 1000 kcal)	1	54.9	17.6	14.7	9.8	0.0040
	2	42.8	18.3	15.8	5.5	0.0267
Veg., potato, and legume intake (100 g)	1	20.6	7.3	14.7	8.0	0.0083
	2	16.9	7.0	15.8	5.9	0.0212
Veg., potato, and legume intake (100 g per 1000 kcal)	1	40.8	17.1	14.6	5.7	0.0239
	2	30.0	16.3	15.7	3.4	0.0754

^†^ Vegetable intake did not include potato and legume consumption unless otherwise noted. Model 1 included statistical adjustment for differences in age and race. Model 2 included adjustment for differences in age, race, BMI, physical activity, smoking, and alcohol use. R^2^ represents the variance accounted for by the full model. Interpretation is as follows: Regarding vegetable consumption, including potato intake, for each 100 g (3.5 ounce) higher intake, after adjusting for differences in age and race, telomeres were 21.2 base pairs longer, on average (F = 7.1, *p* = 0.0123).

**Table 4:** Relationship between fruit and vegetable intake and telomere length in U.S. men only.

Exposure Variable	Model	Regression Coefficient	SE	R^2^ (%)	F	*p*
Fruit and vegetable intake (100 g) ^†^	1	22.4	7.0	19.0	10.1	0.0034
	2	20.4	7.0	19.8	8.4	0.0070
Fruit and vegetable intake (100 g per 1000 kcal) ^†^	1	53.2	15.2	18.8	12.3	0.0015
	2	45.8	15.2	19.5	9.0	0.0054
Fruit intake (100 g)	1	9.3	10.4	18.7	0.8	0.3747
	2	6.7	9.7	19.5	0.5	0.4925
Fruit intake (100 g per 1000 kcal)	1	34.4	24.2	18.7	2.0	0.1661
	2	25.5	22.4	19.4	1.3	0.2649
Vegetable intake (100 g) ^†^	1	37.3	10.5	19.3	12.8	0.0013
	2	35.0	10.8	10.8	10.4	0.0031
Vegetable intake (100 g per 1000 kcal) ^†^	1	102.7	25.4	19.3	16.2	0.0004
	2	93.0	26.4	20.0	12.3	0.0015
Potato intake (100 g)	1	−10.1	14.5	19.0	0.5	0.4909
	2	−9.7	14.2	19.8	0.5	0.5031
Potato intake (100 g per 1000 kcal)	1	−35.3	43.0	18.9	0.7	0.4198
	2	−37.0	42.3	19.8	0.8	0.3893
Legume and pulse intake (100 g)	1	−4.4	14.0	18.9	0.1	0.7570
	2	−7.3	14.6	19.6	0.3	0.6177
Legume and pulse intake (100 g per 1000 kcal)	1	−16.0	34.5	18.8	0.2	0.6456
	2	−27.4	35.9	19.6	0.6	0.4514
Vegetable and potato intake (100 g)	1	13.5	7.9	18.9	2.9	0.0992
	2	12.1	8.2	19.7	2.2	0.1517
Vegetable and potato intake (100 g per 1000 kcal)	1	43.3	20.9	18.8	4.3	0.0463
	2	36.5	22.2	19.6	2.7	0.1107
Veg., potato, and legume intake (100 g)	1	11.2	7.1	19.0	2.5	0.1258
	2	9.1	7.5	19.8	1.5	0.2309
Veg., potato, and legume intake (100 g per 1000 kcal)	1	28.6	18.6	18.7	2.4	0.1362
	2	20.3	20.2	19.5	1.0	0.3239

^†^ Vegetable intake did not include potato or legume consumption unless otherwise noted. Model 1 included statistical adjustment for differences in age and race. Model 2 included adjustment for differences in age, race, BMI, physical activity, smoking, and alcohol use. R^2^ represents the variance accounted for by the full model. Interpretation is as follows: Regarding fruit and vegetable consumption combined (grams), for each 100 g (3.5 ounce) higher intake, after adjusting for differences in age and race, telomeres were 22.4 base pairs longer, on average (F = 10.1, *p* = 0.0034).

## Data Availability

All data supporting the finding are posted online as part of the National Health and Nutrition Examination Survey (NHANES). The data are free and can be found at the following website: https://wwwn.cdc.gov/nchs/nhanes/Default.aspx (accessed on 22 April 2021).
